# Fabrication and Characterization of Degradable Crop-Straw-Fiber Composite Film Using In Situ Polymerization with Melamine–Urea–Formaldehyde Prepolymer for Agricultural Film Mulching

**DOI:** 10.3390/ma15155170

**Published:** 2022-07-26

**Authors:** Qian Lang, Chuanhao Liu, Xiaoxin Zhu, Chao Zhang, Shengming Zhang, Longhai Li, Shuang Liu, Haitao Chen

**Affiliations:** Faculty of Engineering, Northeast Agricultural University, Haerbin 150030, China; liuchuanhao4521@126.com (C.L.); xiaoxinzhu1234@126.com (X.Z.); zhangchao12345@126.com (C.Z.); zsmdblydx@163.com (S.Z.); lilonghai@neau.edu.cn (L.L.); liushuang1988@126.com (S.L.); haitaochen1965@126.com (H.C.)

**Keywords:** biomaterials, biodegradation, mulching film, crop straw, fiber biocomposites, melamine–urea–formaldehyde

## Abstract

Soil mulch composite films composed of biodegradable materials are being increasingly used in agriculture. In this study, mulch films based on wheat straw fiber and an environmentally friendly modifier were prepared via in situ polymerization and tested as the ridge mulch for crops. The mechanical properties of the straw fiber film were significantly enhanced by the modification. In particular, the films exhibited a noticeable increase in dry and wet tensile strength from 2.35 to 4.15 and 0.41 to 1.51 kN/m, respectively, with increasing filler content from 0% to 25%. The contact angle of the straw also showed an improvement based on its hydrophilicity. The crystallinity of the modified film was higher than that of the unmodified film and increased with modifier content. The changes in chemical interaction of the straw fiber film were determined by Fourier transform infrared spectroscopy, and the thermal stability of the unmodified film was improved by in situ polymerization. Scanning electron microscopy images indicated that the modifier was uniformly dispersed in the fiber film, resulting in an improvement in its mechanical properties. The modified straw fiber films could be degraded after mulching for approximately 50 days. Overall, the superior properties of the modified straw fiber film lend it great potential for agricultural application.

## 1. Introduction

Plastic films have been widely adopted in agriculture since the 1960s to increase soil temperature, reduce evaporation, and conserve water in the soil, ultimately improving plant growth [1,2]. Due to their excellent tensile strength and resistance to degradation, the most extensively used materials for agricultural mulching applications are polyethylenes (PEs), such as low-density polyethylene (LDPE) and linear low-density polyethylene (LLDPE), and the copolymer poly(ethylene-vinyl acetate) (EVA) [3,4]. Estimates indicate that 1,950,000 tons/year of plastic mulch are used worldwide, mulching approximately 20 million hectares of land [5,6,7]. In China, estimates indicate that 145 million tons of various types of plastic mulch are used each year [8,9]. However, traditional mulch films are composed of persistent synthetic polymeric materials that are difficult to recycle. The remnant film pieces that form a small fraction of the plastic residues have been given the name microplastics. Accumulated “white pollution” in the soil can deteriorate its structure, reduce nutrient availability and crop yields, and affect soil health and productivity. Based on these considerations, it is important to determine a biodegradable material able to replace plastic mulch and avoid pollution [10,11].

To overcome environmental pollution caused by plastic films, soil-biodegradable materials can be exploited as a potential alternative for use in agriculture. Degradable mulch film is commonly composed of biobased materials that rely on the biodegradable effects of sunlight (ultraviolet rays) and soil microorganisms [12,13,14,15]. Moreover, biodegradable mulch film can be decomposed into carbon dioxide, water, and minerals, which are harmless to the environment. Moreover, the degradation rate of biodegradable films is influenced by the chemical components of the included polymers [16].

Crop straw obtained from corn, rice, wheat, sorghum, soybean, or sugar cane is an abundant source of biomaterials. In China, estimates indicate that 700 million tons of various types of crop straw are generated each year [17]. There are many attempts to use straw for manufacturing wood-based composites by agricultural residues, including straw [18,19,20]. As an abundant agricultural residue, crop straw has a higher ash content that limits its application as fodder but has a potential additional value. Normally, the ash content of agricultural crop straw is from 5% to 15% [17,18]. Currently, a large portion of crop straw is burned directly after reaping, which results in serious environmental air pollution, especially the emission of particulate matter (PM_2.5_ and PM_10_), nitrogen oxide (NO_x_), sulfur oxide (SO_x_), and black carbon (C) [21,22,23]. Therefore, it is necessary to find a replaceable method for the treatment of redundant crop straw [24]. Presently, rice straw, corn straw, soybean straw, and wheat straw are the foremost agricultural residues. Agricultural straw is mainly used as a fertilizer, animal fodder, a fuel, a paper filler, and a mushroom basing material [25]. However, the overall application of agricultural straw is low and varies with the geographic region.

Considerable research has been performed on plastic films, while less attention has been paid to crop straw as a material for film mulch. Compared with plastic and copolymers, different species of crop straw have various weight percentages of chemical components [26]. As the major component of crop straw fiber, cellulose is a linear polymer of (1–4)-linked b-D-gluco-pyranosyl residues, which contain extensive hydrogen bonding. Therefore, combined chemi-mechanical modification leads to chemical changes in the internal surface area and epidermal cells, which can affect the physicochemical characteristics of straw fiber film.

Compared with plastic film, straw fiber film exhibits less effective thermal insulation performance. Many researchers have noted that the influence of plastic film mulch on soil temperature varies during the growing season [27,28,29]. Modification by a chemi-mechanical technique is an effective method to improve the thermal insulation ability of straw mulch film. Additives are also useful to modify its mechanical and thermal insulation performance. In situ polymerization is another effective approach to modification, creating a reaction with the straw fiber that leads to a new chemical structure. At the same time, chemical pretreatment accelerates the digestion of chemical components (cellulose, hemicellulose, and lignin) and the breakdown of the crop straw crystalline structure [30]. This can also improve mechanical performance and degradability.

To provide a practical mulching solution for agriculture, a biodegradable straw mulch film based on crop straw, wood fiber, and environmentally friendly prepolymer for agricultural mulching was designed with melamine–urea–formaldehyde prepolymer using in situ polymerization. The hydrophilicity of the unmodified and modified straw fiber composites films was determined by contact angle measurements. The mechanical characteristics of the films were then investigated. Further, Fourier transform infrared spectroscopy (FTIR), X-ray diffraction (XRD), and thermogravimetric analysis (TGA) were conducted to determine the modification mechanism between the film and the modifier. Finally, the morphologies and elemental plane distributions of the unmodified and modified films were analyzed by scanning electron microscopy with energy-dispersive X-ray spectroscopy (SEM–EDX).

## 2. Materials and Methods

### 2.1. Materials

Mature wheat straw was obtained from Northeast China (Haerbin, Heilongjinag) in September 2020. These were thoroughly washed to remove any extraneous impurities and then dried before use. The final moisture content of the wheat straw was approximately 17%. Sulfate bleached softwood pulp (fiber length = 2.42–2.78 mm; moisture content = 11.5%) was purchased from Ilim Pulp, Saint Petersburg, Russia. The paper pulp specimens were cleaned with distilled water until the water reached a neutral pH. The samples were dried in an oven at 80 °C for 60 h, until the weight was constant, for the subsequent tests.

### 2.2. Chemicals

Urea (99% purity), formaldehyde (mass fraction = 37–40%), melamine (99% purity), ammonia, and film stability agents (NH_4_Cl) were obtained from Tianjin Chemical Reagent Factory (Tianjin, China). All the chemicals were of analytical grade.

### 2.3. Synthesis of the Melamine–Urea–Formaldehyde Prepolymer Modifier

The synthesis process of the melamine–urea–formaldehyde (MUF) prepolymer was based on that in a previous article [31,32]. A three-necked flask (500 mL) was charged with urea (63%, *w/w*), formaldehyde (35%, *w/w*), and ammonia (13%, *w/w*) for the reaction of the urea–formaldehyde (UF) prepolymer. The reaction mixture was vigorously stirred and heated to 39 °C for 4 h. Then, 15% melamine was added to the UF prepolymer and kept at 70 °C for 2 h while stirring. Sodium hydroxide and hydrochloric acid were then added to regulate the pH of the mixture to 8.5–9.0. The MUF prepolymer was next transferred to a plastic container for the following experiment. The physicochemical characteristics of the MUF prepolymer are presented in Table 1. An average of at least three replicate tests were performed for the MUF prepolymer. The pH was measured by a pH instrument (BPH 7100, Bell company, China). The viscosity was tested by a rotary viscometer (NDJ-9S, China). The solid content was carried out according to Chinese Standard GB/T 2793-1995 (Test method for nonvolatile content of adhesives) [33]. The free formaldehyde content was based on the Chinese Standard GB/T 5544-1985 (Resin finishing agent-determination of free formaldehyde content) [34].

### 2.4. Preparation of the Melamine–Urea–Formaldehyde Prepolymer Straw Fiber Composite Films

**Figure 1:**
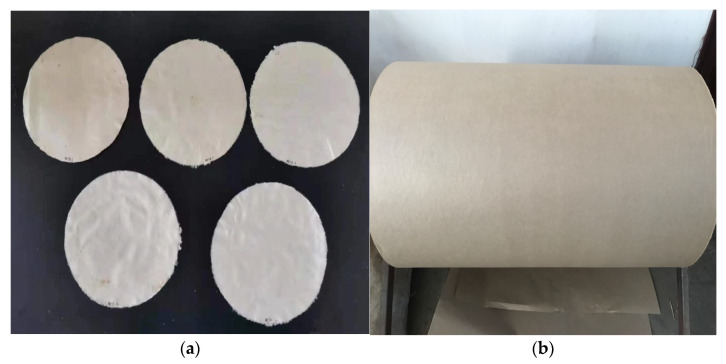
Straw fiber composite film samples: (**a**) experimental paper machine samples and (**b**) large-scale paper-making machine samples.

Residue and excessive dirt were removed from the raw straw fiber by washing with distilled water. The cut wheat straw was then soaked in water for 4 h. Next, the raw wheat straw was chopped using fiber grinding equipment (D-200; self-made by the Northeast Agricultural University) and oven-dried at 70 °C for 2 days. Afterward, straw fiber and paper pulp were defibrated by a Waring blender (ZTG-100, Chang Chun, China) based on the Chinese Standard QB/T 24325-2009. The fabrication of straw fiber film was carried out according to GB/T 7981-1987 (Pulps—Preparation of laboratory sheets: Conventional sheet-former method, China) [35]. The straw pulp, fiber pulp, and chemical agent mixture were stirred at 80 rpm using a mechanical stirrer at room temperature (25 °C). Table 2 lists the formulas of the pulp and chemical agent mixtures. The basic weight of the straw fiber film was 60 g/m^2^. After the solution became homogeneous, it was poured onto a paper-making machine and kept at approximately 97 °C for 6–8 min, until it was completely dry. The experimental paper machine samples and large-scale paper-making machine samples are shown in Figure 1.

Footnote: the mass percentage of the modifier and the chemical stability account for the bone-dry nature of the pulp.

### 2.5. Degradability Experiment

A field experiment was conducted at the Northeast Agricultural University experimental station in Heilongjiang, Harbin (45°44′ N, 126°43′ E), China, from May 2020, to October 2020. For the experiment, soybean was planted in individual holes using a small seeder, and the resulting ridge was covered with modified straw fiber film. Any weeds were removed from the planting area throughout the entire mulching period. Drip irrigation and fertilization were undertaken every week. The field management tasks were carried out based on local farming practices.

### 2.6. Straw Fiber Film Characterization

#### 2.6.1. Mechanical Properties

The mechanical properties of the modified and unmodified samples were measured using a universal testing machine (PinXiang Manufacturing, Hangzhou, China) in accordance with the Chinese Standard GB 12914-2008 (Paper and board—Determination of tensile properties with constant rate of loading approach) [36]. Specimens were tested with a loading velocity of 20 mm/min and a gauge length of 18 cm at room temperature. The straw fiber film was chopped into 10 × 1.5-cm strips. An average of at least five replicate tests were performed for each film.

#### 2.6.2. Contact Angle Measurement

The hydrophilicity of the straw fiber film was calculated by testing the pure water contact angles based on the sessile drop technique using a standard contact angle goniometer (Attension Theta, Biolin Scientific, Goteborg, Sweden). For each measurement, 5 μL of water was pipetted from a syringe onto the film surface. Ten equilibrium contact angles were measured for each film sample, where the average of the left and right plateau contact angles defined the equilibrium contact angle. Images of each solution droplet over time were automatically documented using the computer simulation software. Experiments were operated at room temperature and approximately 65% humidity.

#### 2.6.3. XRD Analysis

The straw fiber film XRD patterns were recorded with an X’Pert PRO MPD diffractometer (Rigaku, Tokyo, Japan) equipped with an X’Celerator detector using a Cu-Kα target (λ = 0.15405 nm). For the experiment, a voltage of 40 kV and a filament current of 30 mA were used. The 2*θ* values were varied from 5° to 50° at steps of 0.05° and a counting time of 60 s.

The crystallinity (Xc) was calculated using the following equation:

(1) $$X_{c}=\frac{A_{\mathrm{cr}}}{A_{\mathrm{cr}}+A_{m}}\times 100,$$

where A_cr_ and A_m_ represented the areas of the crystalline and amorphous regions, respectively [37].

#### 2.6.4. Thermogravimetric Analysis

The thermal properties of the straw fiber film were analyzed by TGA (TA SDT-Q600, New Castle, DE, USA). Approximately 6 mg of the straw fiber film specimens was loosely distributed into an aluminum pan. All the samples were heated from 25 °C to 800 °C at a heating rate of 10 °C/min. The sample gases were swept away at a flow rate of 25 mL/min under a nitrogen (N) atmosphere. The values used were the averages taken from measurements of three straw fiber film samples. The standard deviation was ±0.5 °C.

#### 2.6.5. Fourier Transform Infrared Spectroscopy

The chemical groups and bonding arrangements of the film constituents were analyzed using FTIR (Nicolet is50, Medison, WI, USA). The straw fiber film samples were pretreated in an oven at 80 °C for 10 h before use. The measurements were recorded within a wavenumber range of 4000 and 400 cm^−1^, and the data were recorded at a resolution of 4 cm^−1^ over 32 scans. All the samples were tested in reflection mode. The analysis was performed in duplicate.

#### 2.6.6. Morphology Observation

The morphology of the straw fiber film was visualized using SEM (Hitachi SU8010, Tokyo, Japan). The straw fiber film samples were affixed on aluminum stubs and coated with gold before the tests. Depending on the magnification, an operation voltage of 10 kV was applied to avoid damaging the samples.

Energy-dispersive X-ray spectroscopy (Falcon, CA, USA) was employed to examine the distribution of C, N, oxygen (O), and silicon (Si) elements on the external surfaces of the samples. The samples were sputter-coated with gold and tested at a 20-kV accelerating voltage.

## 3. Results and Discussion

### 3.1. Mechanical Properties of the Straw Fiber Film

The mechanical characteristics of the straw fiber film under dry and wet conditions are presented in Table 3. Mulch film should have sufficient tensile properties to be installed on the soil by machines. From Table 3, it is evident that with increasing modifier concentration (from 0% to 25%), the dry tensile stress increased from 35.21 to 62.28 N. The dry tensile index also increased from 39.12 to 69.20 N·m/g. Overall, samples made by Formula E exhibited the best dry tensile performance. Meanwhile, the wet tensile stress increased from 6.09 to 22.70 N with increasing modifier content. A similar trend was also found for the tensile strength and deformation. The synergistic interaction between the straw fiber film and MUF prepolymer played an important role in reinforcing the straw fiber film. This increase in wet and dry tensile strength may have been caused in part by the favorable distribution between the straw fiber and modifier. As fiber-reinforced composites, the mechanical properties of straw fiber films rely not only on the properties of the fiber-basing material and the chemi-physical additive fillers but also on the interface between the fiber-basing composites and the modifier filler [38]. The results indicate that the straw fiber film had good mechanical properties and could be used in mulching applications. As maintained by the data on the mechanical characteristics, the modified straw fiber film with 20% MUF (Formula E) prepolymer exhibited the best performance. Therefore, these samples were used for the following characterization.

### 3.2. Contact Angle Measurement

**Figure 2:**
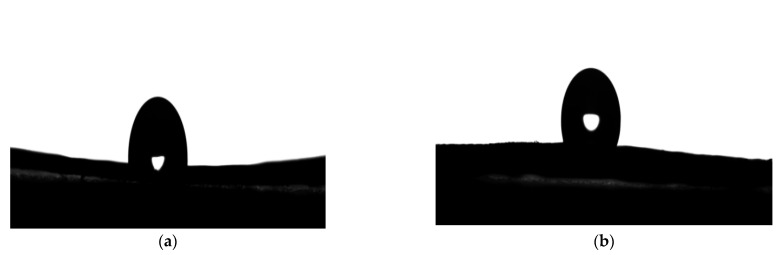
Contact angles of unmodified and modified straw fiber films: (**a**) unmodified samples; (**b**) modified samples.

Typically, the hydrophobic stability of film materials is assessed by water contact angle measurement, where lower hydrophilicity generally indicates a larger contact angle. The water contact angle images of droplets for unmodified and modified samples are shown in Figure 2. It was established that the initial contact angle value of the straw fiber was 95°, indicating that the straw fiber film had poor spreadability regarding the cell solution. After modification, a considerable improvement in the contact angle was obtained (to 117°), providing clear evidence of the super-hydrophobic properties of the modified film. At the same time, it was observed that with increasing concentration of the chemical modifier, the modified straw film became less hydrophilic. It was also revealed that water droplets were quickly adsorbed on the surface of the unmodified straw fiber film, whereas they remained on the surface of the modified film for a much longer period of time. The increased contact angle value observed for the modified film can be ascribed to the formation of a large-scale modifier network based on the –NHCH_2_OH functional groups from the methylolurea and the carbonyl (C=O) from the fiber carboxyl. As revealed by the FTIR spectra, the hydroxyl (OH) groups and amine (NH_2_) groups from the MUF prepolymer groups were polymerized in situ with the OH groups (straw fiber and prepolymer modifier), which were responsible for the decreasing hydrophilicity [39,40].

### 3.3. XRD Analysis

**Figure 3:**
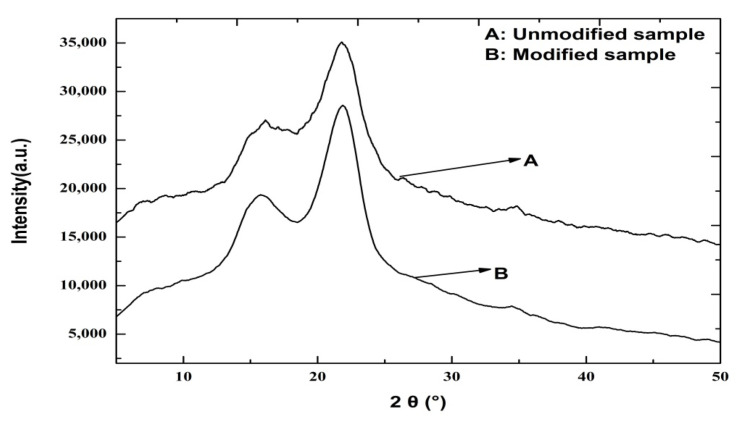
XRD patterns of unmodified and modified straw fiber films.

In general, crystallinity refers to the ratio between a specimen’s crystalline component and its total diffraction [41]. To evaluate the modified straw fiber film quality and the in situ polymerization between the film and chemical modifier, the changes in the straw fiber film skeleton were examined by XRD. The XRD patterns of the unmodified and modified straw fiber films with a 20% concentration MUF prepolymer are shown in Figure 3. Wheat straw is a kind of semicrystalline polymer with characteristic peaks at 2*θ* = 21.5°, 22°, and 23.5°. In addition, the diffraction peak at 18.1° indicates the formation of a highly organized crystalline cellulose structure. Compared with the unmodified straw fiber film, there was no change in the XRD pattern of the modified film, meaning the modifier had only just arrived at the surface of the amorphous and crystal regions. After the addition of the modifier, the crystallinity index of the modified film increased to 60.35% (raw film = 56.62%). The enhancement in the crystallinity of the straw fiber film could be attributable to the formation of chemical bonds between the straw fiber and the OH and amino groups with the modifier cross-linker. An amorphous material has new chemical bonds upon self-assembly. After modification, the molecular interactions between straw fiber films and modifiers improved self-assembly ability which could improve crystallinity [36,37]. In addition, some of the hemicellulose and lignin was removed after modification, which led to increased cellulose rigidity [42]. As a result, the formation of chemical bonds (in situ polymerization) led to a quasi-crystalline structure in the straw fiber film, improving its physical and mechanical performance.

### 3.4. Thermal Properties of the Straw Fiber Composite Film

**Figure 4:**
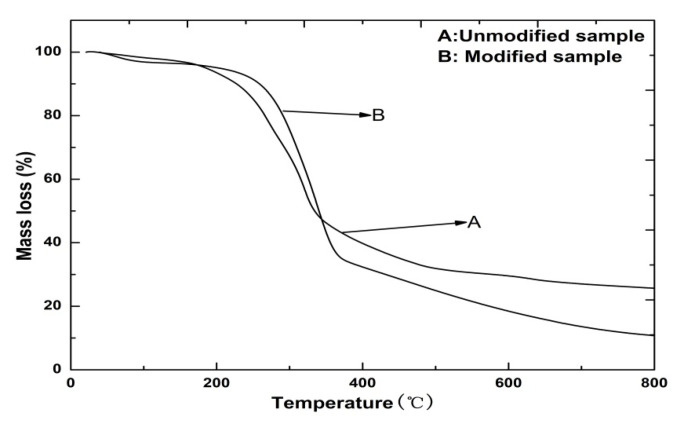
TGA curves of unmodified and modified straw fiber films.

To investigate the variation in the thermostability of the straw fiber film, the TGA results regarding the unmodified and modified films are presented in Figure 4. Based on the characteristics observed during pyrolysis of the straw fiber film, the process as a whole could be divided into three stages. For the unmodified straw fiber films, the first stage occurred from room temperature to 100 °C and was mainly characterized by a 3% mass loss and corresponding weight loss due to evaporation of adsorbed moisture from the fiber, as seen in the TGA curves. In the second stage between 200 °C and 380 °C, severe weight loss occurred due to the decomposition of the film’s major components. The total weight loss percentage at the final temperature (>600 °C) was 71.5%. After modification, the initial decomposition temperature of the straw fiber film increased from 200 °C to 275 °C, indicating the modified films had higher thermal stability than the unmodified ones. In addition, the modified samples demonstrated a larger amount of weight loss due to the removal of calcium oxalate from the lignin and ash after chemical modification [43]. The maximum weight loss temperature changed from 375 °C to 460 °C, indicating the change in the film components (hemicellulose and lignin). The straw fiber film with 30% MUF prepolymer exhibited the highest thermal degradation rate. Overall, the results indicate that the increased thermostability of straw fiber film can be attributed to the reaction between the modifier and fiber. This increase in thermal properties is consistent with results obtained from the XRD analysis.

### 3.5. Fourier Transform Infrared Spectra Analysis

**Figure 5:**
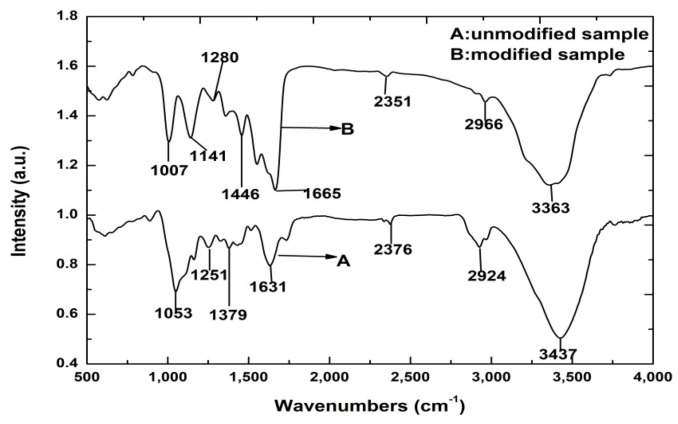
FTIR spectra of unmodified and modified straw fiber film.

Fourier transform infrared spectra analysis is a useful tool for studying the composition of a modifier and its interactions with the compounds in straw fiber film. Figure 5 shows characteristic peaks of the unmodified and modified straw fiber films. Both the unmodified and modified films displayed broad absorption peaks within 3680–3100 cm^−1^, corresponding to the hydrogen-bonded O–H and N–H groups in their polymeric structures [44,45]. The bands observed at 2930–2910 cm^−1^ can be clearly ascribed to the characteristic peaks of C–H, confirming an increase in the number of methylene groups (–CH_2_–) due to the MUF prepolymer. The absorption peaks at 1735–1640 cm^−1^ correspond to the stretching frequencies of various types of C=O groups in the straw fiber film. After modification, the OH stretching vibrations of the modified straw fiber films were observed at approximately 2902 cm^−1^. The amount of –OH decreased noticeably in the modified straw fiber films, indicating the chemically bonding groups formed after modification. Comparing the spectrum of the modified straw fiber film to that of the unmodified film, the appearance of new peaks at 2947 and 2862 cm^−1^ reflect the characteristic –CH_2_– stretching vibrations. The occurrence of a band at 1460 cm^−1^ was the result of the –CH_2_– bending (scissoring) vibration of the silylated alkyl chains in the MUF prepolymer. Further, the peak at 1515 cm^−1^ corresponded to the N–H bending of the NH_2_ group, indicating successful chemical modification of the film with MUF prepolymer. Cross-linking between the MUF prepolymer and the straw fiber film led to the formation of C–O bonds. The existence of the band at 1280 cm^−1^ can be attributed to the formation of chemical bonds between the MUF prepolymer and the film. The peaks at 1664 and 1548 cm^−1^ were derived from the triazine ring of the MUF prepolymer. Overall, the detection of different chemical bonds in the unmodified and modified films revealed chemical cross-linking between the film fibers and MUF prepolymer.

### 3.6. Scanning Electron Microscopy Analysis

**Figure 6:**
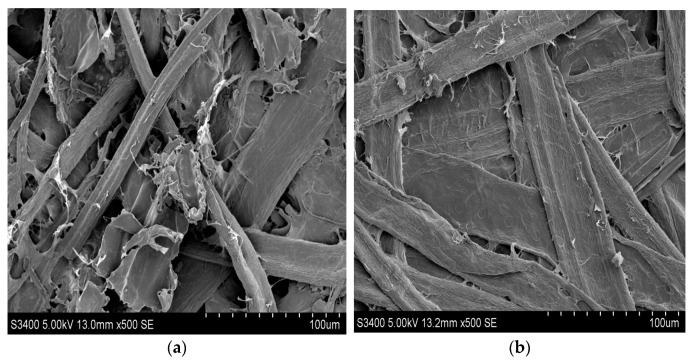
SEM photograph of straw fiber film morphology: (**a**) unmodified and (**b**) modified straw fiber film.

The surfaces of the unmodified and modified straw fiber films were examined by SEM, as illustrated in Figure 6. The addition of the modifier noticeably altered the surface morphology of the film. The images revealed clear differences between films with and without modification, with the unmodified film exhibiting higher roughness compared with the modified film, which presented a smooth and compact surface. In the modified film, the modifier was homogeneously spread on the surface of the straw fiber film. It appears that as the modifier content increased, a dense layer structure became observable. This indicated that a strong interaction occurred between the chemical modifier and the straw fiber, which may have been due to the strong intermolecular interaction, resulting in significant improvement of the film’s mechanical properties [46].

### 3.7. Energy-Dispersive X-ray Spectroscopy

**Figure 7:**
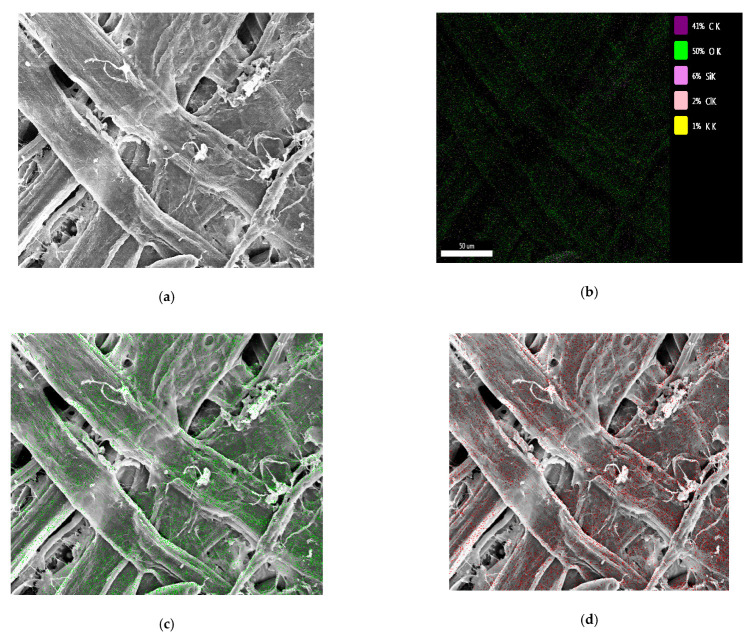
EDX analysis of the straw fiber film: (**a**) SEM of modified straw fiber film; (**b**) distribution of C, O, Si, chlorine (Cl), and potassium (K); (**c**) distribution of O; and (**d**) distribution of C.

Quantitative analysis of various atoms was performed by EDX. The EDX micrographs (Figure 7) presented the dispersion–aggregation phenomena of the C, O, and Si on the surface of the straw fiber film samples. The different color spots over the dark background represent the locations of the relative elements on the external surface of the straw fiber film. The statistics reveal that the C, O, and Si contents were 41%, 50%, and 6%, respectively. Further, the EDX spectrum of the modified straw fiber film exhibited a uniform distribution of O and Si, indicating successful in situ polymerization between the modifier and the film. The good dispersion of the modifier in the straw fiber film also confirms the homogeneity of the modifier–film interaction.

### 3.8. Degradability of the Modified Straw Fiber Film

**Figure 8:**
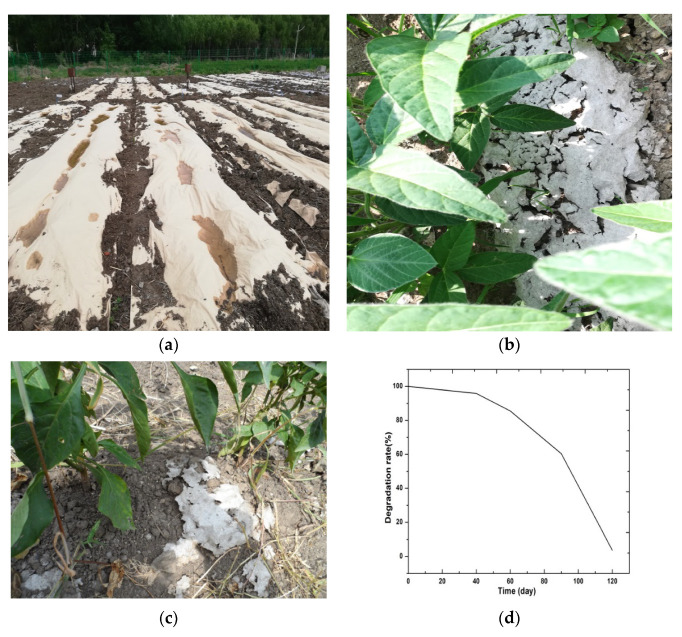
Degradability experiment on the straw fiber film: (**a**) ridge mulching with modified straw fiber film; (**b**) cracking period of the modified film; (**c**) the modified film after degradation; (**d**) degradation rate of modified film.

The degradation process of the straw fiber film included the following stages: (1) the appearance of tiny pores, (2) the appearance of huge pores and large-area rupture, and (3) breaking into small pieces [47,48,49]. The process, which led to thinning, brittleness, and loss of mechanical properties, is shown in Figure 8. The results demonstrated that the modified straw fiber film began to develop small cracks approximately 40 days after mulching. The degradation rate of the biodegradable film accelerated at approximately 60 days. A large area of rupture and degradation then appeared at approximately 90 days, at which point some parts of the film buried in the soil were almost fully degraded. The degradation area also reached 60% at approximately 90 days. After 120 days, the modified straw fiber film was completely degraded. Overall, the modified film gradually degraded after mulching due to the environmental effects of soil microorganisms, enzymes, oxygen, temperature, and water. Compared with traditional PE, the modified straw fiber film underwent rapid degradation in soil. Accordingly, this film can be used as a mulching film for crops with short growth periods, such as strawberries, tobacco, garlic, or rice.

## 4. Conclusions

This study investigated the properties and characteristics of degradable crop-straw-fiber films modified by in situ polymerization with MUF prepolymer for agricultural film mulching. The addition of MUF prepolymer resulted in notable improvements in the film’s mechanical characteristics, especially the tensile properties. After modification, the contact angle of the modified straw fiber films was increased, indicating a decrease in the hydrophilicity. The XRD patterns of the samples were not manifestly changed, while the relative crystallinity increased, confirming an improvement in the mechanical properties of the films. Thermogravimetric analysis indicated that the MUF prepolymer caused a remarkable increase in the thermal stability of the films. The FTIR spectra also revealed the formation of a new chemical bond between the MUF prepolymer and the films. The results of the SEM–EDX indicated that the chemical modifier was dispersed well in the crop-straw-fiber film and had good adhesion in the interfacial area due to hydrogen bonding. The modified straw fiber films were also found to have a much shorter degradation time, making them appropriate for application to crops with a short growth period. According to the results, MUF prepolymer can be used as a suitable environmentally friendly chemical additive for enhancing the characteristics of degradable crop-straw-fiber film. The superior properties of the resulting modified films make them good candidates for agricultural applications.

## Figures and Tables

**Table 1:** Physicochemical properties of the MUF prepolymer.

Name	Density (g/cm^3^)	pH	Viscosity (mPa s)	Solid Content (%)	Free Formaldehyde Content (mg/100 g)
MUF	1.30	8.2	13	59.60	0.07

**Table 2:** Formulas of straw fiber film modification.

Name	Wheat Straw Pulp (%)	Bleached Softwood Pulp (%)	MUF (%)	Chemical Stability A (%)	Chemical Stability B (%)
A	40	60	0	0	0
B	40	60	5	2	3
C	40	60	10	2	3
D	40	60	15	2	3
E	40	60	20	2	3
F	40	60	25	2	3

**Table 3:** Mechanical properties of the unmodified and modified straw fiber films.

**Name**	**Dry Tensile Stress (N)**	**Deformation (mm)**	**Tensile Strength (kN/m)**	**Tensile Index (N·m/g)**	**Basis Weight (g/m2)**	**Breaking Length** **(mm)**
A	35.21	1.93	2.35	39.12	60	15
B	40.97	1.97	2.73	45.52	60	15
C	43.07	1.78	2.87	47.86	60	15
D	55.92	2.09	3.73	62.13	60	15
E	62.28	2.36	4.15	69.20	60	15
F	57.14	3.01	3.61	60.16	60	15
**Name**	**Wet Tensile Stress (N)**	**Deformation (mm)**	**Tensile Strength (kN/m)**	**Tensile Index (N·m/g)**	**Basis weight (g/m2)**	**Breaking Length** **(mm)**
A	6.09	2.51	0.41	6.77	60	15
B	13.25	4.12	0.88	14.72	60	15
C	16.96	4.96	1.13	18.84	60	15
D	18.88	5.27	1.26	20.98	60	15
E	22.70	5.34	1.51	22.13	60	15
F	19.78	5.15	1.32	21.98	60	15

## Data Availability

All data included in this study are available upon request by contact with the corresponding author.
